# Engagement in assertive community treatment as experienced by recovering clients with severe mental illness and concurrent substance use

**DOI:** 10.1186/1752-4458-8-40

**Published:** 2014-10-31

**Authors:** Henning Pettersen, Torleif Ruud, Edle Ravndal, Ingrid Havnes, Anne Landheim

**Affiliations:** National Centre for Dual Diagnosis, Innlandet Hospital Trust, P.O. Box 104, N-2381 Brumunddal, Norway; SERAF – Norwegian Centre for Addiction Research, University of Oslo, Kirkevn.166, N-0407 Oslo, Norway; Division Mental Health Services, Akershus University Hospital, N-1478 Lørenskog, Norway; Institute of Clinical Medicine, University of Oslo, P.O. Box 1171, N-0318 Oslo, Norway; Division of Mental Health and Addiction, Oslo University Hospital, P.O. Box 4956, N-0424 Oslo, Norway

**Keywords:** Severe mental illness, Substance use, Assertive community treatment, Client experiences, Engagement, Qualitative study

## Abstract

**Background:**

Clients with severe mental illness (SMI) who use substances are less engaged in treatment than those who do not use substances, and assertive community treatment (ACT) engages and retains clients with SMI and concurrent substance use at a higher rate compared with traditional treatment. This qualitative study aimed to explore the experiences of being recruited to, and remaining in, ACT among recovering clients diagnosed with SMI and concurrent substance use.

**Methods:**

Twenty semi-structured interviews were undertaken among 11 clients with SMI and concurrent substance use who were included in ACT teams. The inclusion criteria were SMI and concurrent substance use and improvement after a minimum of 12 months in treatment regarding one or several of the following parameters: quality of life, general functioning and substance use. Systematic text condensation was applied in the analyses.

**Results:**

The experiences of building trust through enduring involvement and receiving benefits were most important for the acceptance of ACT by clients. A feeling of exclusiveness, perceiving ACT as a safety net and the clients’ own personal responsibility for taking part in the treatment were stated as the most important factors for remaining in treatment.

**Conclusions:**

The implications of the results of the present study are that service providers have to prove that they can be trusted in the initial phase of the clients’ contact with the team. The feeling by clients with SMI and concurrent substance use that service providers in ACT believe they can improve their client’s quality of life, is of importance for feeling exclusive, having hope for the future and remaining in treatment.

## Background

Assertive community treatment (ACT) is a team-based, service-delivery model for providing comprehensive community-based treatment to clients with severe and persistent mental illnesses who did not benefit from traditional treatment [1]. The treatment model was first described as training in community living [2]. A Cochrane review concluded that ACT (compared with standard treatment) reduces the frequency and duration of hospitalization, secures better housing and leads to improved client satisfaction; moreover, clients were more likely to remain in contact with services [3]. Both epidemiological [4] and clinical [5] studies have shown that clients with severe mental illness (SMI) are more likely to use substances compared with others. The ACT model has been developed to include persons with problematic substance use concomitant with SMI [6, 7], and integrated dual disorder treatment is one of the working tools in a regular ACT team.

The concept of engagement has attracted little attention in clinical research. Engagement has been defined as a complex phenomenon that encompasses factors that include acceptance of a need for help, the formation of a therapeutic alliance with professionals, satisfaction with the help already received and a mutual acceptance of and working toward shared goals [8]. The REACT study of Assertive Outreach Teams in London [9] found that clients were better engaged in ACT compared with traditional treatment. Clients who misused substances were less well engaged than those who did not misuse substances [10]. Research also indicates that assertive outreach treatment is able to engage and retain clients with SMI and substance use at a higher rate compared with traditional treatment [11].

The ACT model emphasizes the following key principles when recruiting clients into treatment: collaboration, motivational interventions to engage clients and building intrinsic motivation for receiving services from the team. Where necessary, therapeutic limit-setting interventions are used to create extrinsic motivation for receiving services that are deemed necessary to prevent harm to the client or others [1, 12]. Research that examined the accounts of clients’ experiences of ACT indicates that social interventions and acceptance of clients’ world views stimulated engagement [13]. The same authors performed a study of the processes of clients’ engagement in, and disengagement from, ACT. The most important factors were time and commitment of the staff, social support and engagement without a focus on medication and a partnership model of the therapeutic relationship [14]. One study examined the perceptions of both clients and staff of the techniques that are used to encourage adherence to treatment. Both groups reported that supporting clients and building relationships were the preferred mechanisms for promoting treatment goals [15]. A more recent study on the perceptions of clients and staff regarding engagement in ACT found three major themes as being most significant for both groups: providing and receiving practical assistance, having a genuine two-way conversation and valuing the experiences and personal attributes of the other person [16].

Research on the perceptions of engagement in ACT by clients with SMI and concurrent substance use is limited. One case study identified the persistence demonstrated by service providers and the trust that clients developed in their service providers as primary factors for successful engagement [17]. One study of clients’ perceptions of first-year experiences of treatment in ACT found that service delivery in a caring manner, persistence and aiding in practical matters were most important for engagement [18]. This manuscript attempted to address a gap in the literature by exploring clients’ experiences of engagement in ACT. The study aimed to explore experiences of being recruited to, and remaining in ACT by recovering clients diagnosed with SMI and concurrent substance use.

## Methods

The study design was descriptive and explorative, and used a phenomenological approach that aimed to examine the participants’ experiences. A purposeful and criterion-based sampling procedure [19] was considered appropriate for our study. The intention was to maximize the advantages of in-depth, purposeful sampling to lessen the influence of a small sample size. To allow sufficient time for each individual to provide the necessary in-depth information, we chose to perform individual semi-structured interviews with a focus on each participant’s life world [20]. Transcriptions of the interviews were analysed using systematic text condensation [21], which is a stepwise procedure influenced by phenomenology [22, 23] that is well suited to examining life-world experiences.

### Recruitment and setting

Through initiatives from the Norwegian health authorities, 12 ACT teams were established in Norway between 2008 and 2011 to try out this model of service delivery. The Norwegian ACT teams were established 1–4 years before the interviews were conducted, and we chose to establish contact with the teams who had clients with the longest duration of engagement in ACT. Accordingly, the five teams who had been operating for 2–4 years were selected. Otherwise, the chosen teams did not differ from the other teams. In total, 349 clients had been included in the 12 ACT teams by the time of the interviews. About 60% of the clients had problematic substance use together with severe mental illness. This implied that about 200 clients could be characterized as having a dual disorder. We did not know exactly how many of these clients had been in treatment for more than 12 months or how many of these clients were in recovery. Eleven clients from five different ACT teams located in urban and suburban areas of Norway were recruited into the study, with 1–3 clients from each team. Contact was first established via telephone conversations with the team leaders. This contact was followed by an email explaining the purpose of the study, and a request for permission for the first author to interview clients who met the inclusion criteria for the study: clients with SMI and concurrent substance use treated in ACT teams who, after a minimum of 12 months of treatment, had made progress according to both the client and the team in terms of their quality of life and/or general functioning and/or substance use. The team leaders recruited the participants by consulting the other members of the team regarding whether they had patients who met the inclusion criteria for the study and who were both willing and able to go through interviews. If some of the participants were in poor condition at the actual time of the interview, we made agreements with the team leader and the participant to perform the interview with a therapist attending. The team leaders also made appointments for the interviews. We do not know exactly how many patients were asked to participate or how many refused to take part in the study. However, our impression was that most of those who were asked agreed to participate.

### Participants

Eleven participants (nine men and two women) aged 27–63 years (mean, 39 years) were included in the study (Table 1). The duration of ACT was 14–30 months (mean, 22 months) at the time of the first interview. Most clients had a diagnosis of schizophrenia or schizoaffective disorder; however, clients who were diagnosed with bipolar disorder or an unspecified psychotic disorder also participated in the study. For most participants, SMI had preceded substance use. The substances used were mainly cannabis and amphetamine and, to a lesser extent, alcohol. Cocaine and prescription drugs, such as benzodiazepines and codeine, were seldom used. Most participants used multiple substances. Few used substances on a daily basis. The most typical use was 3–4 times a week, often in connection with a worsening of the mental illness [24]. Several participants reported a troublesome childhood supported by child welfare authorities. For all participants, the treatment setting represented a new arena, as ACT is novel in Norway. The majority of the participants had less than 2 years of experience with ACT, but all had histories of several years of receiving traditional psychiatric and substance-use treatment. Hence, the participants were able to describe comparisons between ACT and traditional treatment. Only one participant was on forced medication because of long periods of instability in relation to mental health, substance use and antipsychotic medication. Similarly, another participant was hospitalized involuntarily between the interviews.

**Table 1:** **Psychiatric diagnoses, self-reported substance use and duration of treatment in ACT**

Participant	Age	Psychiatric diagnosis (ICD-10)	Self-reported substance use	Duration of treatment in ACT (months)
P1	42	Hebefrenic schizophrenia	Alcohol, cannabis	30
P2	46	Paranoid schizophrenia	Cannabis	28
P3	27	Undifferential schizophrenia	Amphetamine, cocaine, cannabis	30
P4	32	Paranoid schizophrenia	Alcohol, cannabis	17
P5	34	Paranoid schizophrenia	Amphetamine, cannabis	19
P6	33	Paranoid schizophrenia	Amphetamine, cannabis	14
P7	30	Paranoid schizophrenia	Amphetamine, cannabis	21
P8	39	Residual schizophrenia	Alcohol	22
P9	42	Bipolar affective disorder	Amphetamine, cannabis	20
P10	38	Psychosis with delusions	Amphetamine	20
P11	63	Paranoid psychosis/delusions	Prescription drugs	19

### Data collection

In total, 20 individual interviews were conducted with the 11 study participants. The interviews lasted from 45 to 75 min, and all but one were recorded digitally and transcribed verbatim. An interview guide with specified topics was used to focus on the relevant experiences of the participants, including the influence of substance use, empowerment and relation to service providers. The main question in the interview guide was: “What was most important for your improvement after joining the ACT team?” As outlined in the analysis section, six overarching themes were established based on the participants’ responses. Service providers in ACT were stated as one of the important factors for improvement, and the follow-up questions were: “What are your experiences of being included by the ACT team?” and “What has been most important for you to remain in treatment?”

The interviews were conducted by the first author predominantly on a one-to-one basis in meeting rooms provided by the ACT team. The intention was to let the participants reflect freely on their experiences and to ask clarifying questions without making interpretations [20]. Two participants were accompanied by their therapist, for safety or support reasons. Nine participants were interviewed twice, with a 5–8-month interval between the interviews. One participant did not agree to a second interview, and another was not accessible because of a worsening of their mental illness. The reason for performing the second interviews after 5–8 months was that, although our intention when planning the study was to interview the participants once, when we completed the first set of interviews, we realized that some issues in the interview guide were not fully covered, and that some of the participants obviously could contribute more data than we had obtained in the first interview. This strategy was chosen instead of recruiting more participants for the study. Furthermore, re-interviewing the participants provided the possibility of having the participants reflect on their experiences over time. Performing a second interview also served as member checking [25], by letting all of the participants read the main points from the transcription of the first interview. In both the first and the second set of interviews, most, but not all, of the transcription was undertaken before the next interview. Between the first and the second interview with each participant, transcription was completed. The participants reported mostly on positive, but also on negative changes in mental state and general functioning between the interviews. None of the participants reconsidered or contradicted what he/she had discussed during the first interview.

### Analysis

To address the aim of the study, qualitative data from the semi-structured interviews were analysed using systematic text condensation [21]. This method is recommended for descriptive and explorative analyses of a phenomenon in reports from different participants and for developing new descriptions of a phenomenon. The interview transcripts were read with an open mind as a means to bracket the researchers’ preconceptions and to focus on what the participants conveyed. By having the ACT teams recruit patients for the study, the first author (interviewer) was only aware of the sex and psychiatric diagnosis of the individuals, and that participants had a substance-use problem when included in the team. The first author was trained as a clinical nurse and had a master’s degree in health promotion, focusing on factors that influence improvement and well-being.

Initially, the transcribed text was read as a whole to derive an overall sense of the data. From this material, six overarching themes were established: *substance use as a coping strategy*, *experiences of abstaining from substance use*, *control and stability*, *socializing*, *active participation* and *service providers in ACT.* This last overarching theme was further elaborated. The second step was the identification of expressions in the dataset that were more informative than the initial codes that were developed during the early reading. The identified meaning units covered all the expressions used by the participants about ACT and constituted the basis for further analysis. During the third step, the following sub-themes were established: *building trust through enduring involvement*, *receiving benefits*, *exclusiveness*, *safety net* and *personal responsibility.* A text of condensed meaning was constructed for each sub-theme. Finally, the descriptions of the phenomena were revised.

The first author performed the interviews, transcribed the interviews, did the initial coding and identified meaning units. In addition, both IH and AL took part in the analysis by identifying initial codes and discussing the emerging themes. The NVivo10 software was used to output all codes to the worksheets, as well as to manage all data and to confirm that no overlapping of data existed within each sub-theme.

### Ethical considerations

The study was approved by the Regional Committee for Medical and Health Research Ethics, South-East Region (no. 1196, 2010) before the clients were recruited. Each participant gave written informed consent to take part in the study and was informed that he/she could withdraw from the study at any stage. The interviews were conducted in meeting rooms provided by the ACT team. Two participants were accompanied by their therapist, which served as a safeguard in the sense that the interviews could elicit emotional reminiscing about sensitive issues. Allowing the team leaders to receive a written or verbal report of the progress of each interview served the same purpose, and provided information on practical matters, such as how long the interview lasted, the need for breaks and whether the participant felt comfortable after having finished the interview. It was important to emphasize that the intention of the interviews was research and not treatment. To provide anonymity, numbers instead of names are used when citing participants, and no recognizable information about the participants is revealed in the article.

## Results

The results of the study mainly emphasized how the study participants viewed important aspects of service providers’ contributions to engaging clients in ACT. The factors that the participants themselves envisioned as their own contributions to their engagement in the same service were also perceived as important, focusing on relational factors. The participants’ experiences are reported on a timeline from the initiation of ACT to the time of the interviews, in accordance with the following themes and sub-themes: the first main theme, *Initial engagement*, included the sub-themes *Building trust through enduring involvement* and *Receiving benefits.* The second main theme, *Maintained engagement*, contained the sub-themes *Exclusiveness*, *Safety net* and *Personal responsibility*.

### Initial engagement

The initial engagement in ACT was described by the participants either as a transfer from institutional treatment or as a gradual process of recruiting clients in their living environments who were not currently engaged or were poorly engaged in other treatments. Furthermore, inclusion in ACT mostly involved a process of gradual acceptance of an unknown treatment. Building trusting relationships through time and receiving benefits had the greatest impact on accepting treatment.

#### Building trust through enduring involvement

The majority of the study participants were previously diagnosed with paranoid schizophrenia and displayed scepticism toward the service providers who started to show up at their homes. Looking back on the time prior to ACT, many participants described experiences of illness and isolated lives together with struggles to manage daily activities. When ACT entered the scene, the participants started receiving daily follow-up with medication and aid in practical matters. This was described by several participants as a gateway into treatment. Resistance to treatment was prominent in the inclusion stage; however, with time, there was a tendency to join what the team had to offer by becoming more open and receptive. For several of the participants, this receptiveness was explained by a gradual awareness of the service providers’ good intentions. Participant 3 had been engaged in poly-substance use since his early teens and had been abstinent from substance use for some months, and he explained that he was in a bad phase of his mental illness when he was contacted by the service providers. He reflected:

*‘I stayed mostly at home and became sick. So, when they [ACT] showed up something started to happen. I received medicines once a week. I saw they were eager to help, despite my bad condition when they entered the scene. The persons working in ACT are all right, and they are nice. They did not give up, and they put a lot of effort into it. I was not so co-operative in the beginning. I did not manage anything, and did not know what to do’.*

Several of the study participants stated that the service providers visited them once or twice a week, even if they had previously rejected them. They kept conveying what they had to offer in positive terms, without being rude or intrusive. Descriptions of the service providers as being persistent and patient were prevalent. The time dimension and the offering of services in a caring manner seemed to be essential both for developing trust toward the new service providers and for building motivation for treatment. A common theme was the involvement displayed by the team; however, some participants reported experiencing a sense of control and surveillance that sometimes accompanied the treatment in the early phase of engagement. With time, these experiences seemed to be smoothed out, leading to the view of involvement as being mainly positive. It seemed as if the participants mostly experienced that someone was listening when the service providers launched strategies for taking prescribed medication. This issue was perceived by some as the most important difference between ACT and traditional in- and out-patient treatment, and of importance for building trust. In contrast, other participants also acknowledged the structure and predictability represented by institutional treatment. The participants had few experiences of coercion or limit-setting strategies in ACT, but they envisioned that coercive means could be used in cases involving lack of co-operation. Discussion of medication reflected an issue on which some of the participants expressed criticism toward ACT. As participant 7 explained:

*‘In my opinion, as long as I seem to be functioning well, from the perspectives of both people outside and the service providers, I should be in charge of my medication. The treatment involves excessive doping. For example, to pull down your pants and show your bottom in front of a youngster in order to receive an injection … that is humiliating. But I’ve learned that opposing leads to more regulations ….’*

Participant 9 was diagnosed with bipolar disorder and had a history of numerous admissions to psychiatric hospitals. He experienced a sense of intrusion and surveillance by the team during the initial phase; however, gaining experience with the service providers changed his attitude:

*‘In the beginning I thought they [ACT] were too involved. But after a while it somehow smoothed out … concerning both positive and negative aspects. Now, I have mostly positive experiences with ACT, because I realize that they are people of good will. That ACT is not a kind of surveillance, but rather treatment. But it can feel like surveillance’.*

The fact that repeated meetings with the service providers entailed attachment was experienced by six of the participants. This resulted in perception of the contact as being more personal than professional. Predominantly, having access to ACT over a prolonged period was expressed as being essential in the process of establishing a positive relationship with service providers. Scepticism toward ACT was associated with some of the participants’ feelings of being under surveillance by the team, or the appreciation of home as an arena free of treatment. It was important to have a place where the client could stay by himself/herself and just relax when they were sick and tired, although this view was only expressed by two participants. Some participants found the treatment provided during the initial phase unfamiliar and somewhat surprising. In addition to having a mental disorder and a substance-use problem, participant 11 was physically ill, expressed being a “loner” and described the inclusion in ACT as follows:

*‘ACT showed up when I was discharged from hospital. They contacted me afterwards. I was very sceptical and thought it was some kind of trick. Eventually I realized it was a kind of self-help offer. I really appreciated it, because then I had someone to lean on. I felt so alone with my problems … I would sit by myself all day long. It took some time before I got to know them and viewed them as supportive. It was totally strange for me and unrealistic that someone should give me support. It was so unexpected’.*

Providers of outreach treatment can also be viewed with suspicion when taking into account the many experiences of unsuccessful treatments that the study participants had endured in the past. Participant 11 was used to struggling alone with mental, physical and substance-use problems. ACT represented a novelty for this participant. Several months of treatment were needed before this participant was able to trust the service providers.

#### Receiving benefits

The majority of the participants emphasized the importance of having someone to lean on when trying to keep up with bureaucratic challenges. The search for stable housing and work possibilities was perceived as both important and demanding. For a large part of the cohort, the decision to accept ACT depended upon enticements that comprised the allocation of benefits. Receiving an offer of a new apartment was the main reason given by some participants for accepting inclusion in ACT. Participant 10 was hospitalized for substance-use treatment at the time of the interviews, and had managed to abstain from substance use for several months. Thinking back, he had a clear vision of what was most important for his improvement:

*‘At that time I had a lot of problems. Then he [ACT team leader] knocked on my door, saying: “I offer you an apartment.” I thought, is it God who sent him, or what? That was when I was hospitalized for substance-use treatment. This I can’t believe, I thought. But it was a fact. He really had an apartment for me. That was the first step, and I felt it as something exceptional’.*

The exceptional nature of the situation included the surprise of facing a service provider offering him improved housing. He described this as being almost an epiphany, or at least the first step of beginning to trust the service providers in ACT. The perception of ACT members as agents who provided initiatives for them to start managing practical matters was prominent among the participants’ descriptions. The service providers were seen more as catalysers and supervisors than as practical workers. Three of the participants described this approach as an aid in self-awareness. Despite several set-backs and a long history of hospitalization, participant 4 had his own apartment, and planned a re-entry into working life:

*‘When ACT started visiting me I felt I got the support I needed. Furthermore, they gave me the kick to manage to sort out things on my own. ACT helped me in talks when meeting the bureaucracy. They stood by me, and pushed to get things through’.*

However, the options that were offered by the service providers proved to be counter-productive if the client found them humiliating or unsuitable. An offer of improved housing had an inclusive effect on one of the participants. In contrast, the offer of a cup of coffee by the service providers in a café caused resistance and suspicion in another participant, who explained it as unproblematic to accept an offer of professional origin, whereas a contribution from private sources was experienced as more of a challenge, almost as an insult. After the service providers explained that the coffee was financed by the ACT funding, the participant felt more comfortable.

### Maintained engagement

The study participants perceived important requirements for remaining in ACT mainly as being embedded in the strategy of the service providers, but to some degree as also being dependent on personal attitudes and the behaviour of the participants themselves. Clients’ experiences of recognition by the service providers as individuals with strengths and weaknesses and of being treated with respect, together with a feeling that service providers could offer assistance during periods of worsening mental illness or substance use, were crucial for adherence to treatment. In addition, there was a sense of the importance of the participants’ own commitment to remain in treatment.

#### Exclusiveness

The study participants experienced that the service providers were able to see them as individuals who deserved treatment, and not just as part of their job. Embedded in this perception was a sense of being pursued, treated respectfully, or seen as a chosen individual. This was not because the participants had a feeling of being superior to others, but because they experienced a greater sense of worth in ACT compared with traditional treatment, which explains the feeling of exclusiveness experienced by the participants. This was exemplified by participant 7, who had a long-term experience of both psychiatric and substance-use treatment and felt that it was important that service providers believed that there was a potential for improvement in relation to both mental health and substance-use problems:

*‘One difference between ACT and traditional treatment may be that they [ACT] treat those they feel like treating. They can select which patients they shall include in treatment on the basis of conversations in advance. At least it feels like it. In a way, I feel like a chosen one, or a person in whom they see possibilities for improvements’.*

The participants perceived the service providers as friends or associates just as much as professionals, and some expressions even reflected the view that ACT had become a small family for a few of the participants. Meetings with the service providers were seen as commonplace and ordinary, which seemed to render communication easier and more straightforward. Participant 1 explained that he had suffered from bullying in his youth, with subsequent isolation and experiences of anxiety and depression. He adopted the family concept in his description, and emphasized the different roles played by the team, and how it kept him in treatment and enabled him to imagine a future:

*‘Most people are concerned with how things are with family and friends. That’s how it is, and for me ACT functions as a kind of family, making phone calls and taking care of things. It contributes to believing in the future’.*

After a while, most of the participants allowed the service providers to enter their home arena, both for verbal communication and helping in practical ways, or simply just for spending some time together. Moreover, receiving services in the home arena clearly counteracted the isolation that many of the participants expressed. This seemed to be prevalent while speaking about the outreaching nature of the organization of the work, although a few of the participants were reluctant to receive treatment in their home. Some participants emphasized difficulties in communicating with family or friends during periods of severe symptoms of their mental illness, and found that ACT was better suited because they had a higher tolerance level regarding problematic matters.

#### Safety net

The study participants shared some of their experiences of anxiety, depression and general discomfort, which could be related to periods of intense substance use. An awareness of future set-backs was expressed by most of the participants based on their previous experiences of living with SMI. However, contact with the ACT team was perceived as a possible way to manage life through different challenges. ACT was described by several participants as an agency that enabled them to live better with anxiety and depression. In a sense, the team was able to take over some of the responsibility, thus making it possible to obtain relief from some of the heavy burdens of living with SMI and substance use, which could worsen in the future. Participant 1 described feelings of relief, comfort and being offered treatment options by staying connected to ACT:

*‘People are important to me. Especially, I experience the relation to ACT as a relief from anxiety and negativity in myself. I can put some of the burdens on them [the ACT team], because they are available. If you become psychotic or suffer a relapse, you have the right to receive treatment’.*

Furthermore, participant 1 described the years spent living with SMI, substance use and challenging life events as a “container” of bad feelings experienced that were much larger than the ACT team could understand or sort out, and that this was something with which he needed to live. To him, not being able to foresee or work out when the container was overflowing and the anxiety was approaching was a challenge. The knowledge that the service providers would show up regularly and his awareness of the presence of the team in the background made him feel safe. Experiences of substance use and psychotic episodes together with admissions to and discharges from institutional care were prevalent in several of the participants’ statements. With reference to these challenges, they appreciated the steadiness represented by ACT. Participant 3 had been abstinent from substance use for some months, but still felt vulnerable and in need of support:

*‘It is essential for me to have some support … like ACT. I will go on with it [ACT], even if I manage to stay clear of substance use. I will also use medicine, because it is good for me. You don’t need to be into substance use to get problems. When I get problems, or I get worse, then it feels safe to have someone behind me’.*

When struggling with isolation and poor social networks, as the majority of the study participants did, they welcomed both the stability and the flexibility provided by the ACT team. In addition, they had come to know the service providers, so that making a phone call during a crisis was less of a barrier compared with what they had experienced in other out-patient services. In this respect, safety became important for reducing the participants’ vulnerability and as a motivation for further treatment.

#### Personal responsibility

Some of the participants made a point of not taking for granted the help they received from ACT. It was important for them not to forsake the service providers. To put them down would imply putting themselves down. They felt obliged to retribute the service via their loyalty toward the service providers. In a sense, this attitude took the form of an act of reciprocity that became a motivation to stay in treatment. Participant 10 had kept regular contact with ACT during his last two institutional stays, and expressed the following feelings:

*‘I was hospitalized for one month for substance-use treatment. Then I was transferred to the current institution. I have not used substances since then. I have always thought that after all the effort they [ACT] have put into it … everything they did for me … I must not take it for granted. They have done a lot for me, so my thought is not to disappoint them. That has been a motivation. If I disappoint them, then I disappoint myself as well’.*

Several participants also described following up on messages and keeping appointments with the service providers as prerequisites for staying in treatment. This implied that matters needed to be taken seriously, thereby ensuring that the team received positive feedback regarding their efforts. Furthermore, there were also statements of the importance of behaving well during contact with the team, as exemplified by participant 1, who expressed his fear of being rejected by the team because of “bad” behaviour:

*‘Slowly, I have learned to know them [the ACT team]. What they look like, when they show up, and the sound of their voices. If they are nice or … or if I have been talking a lot of shit, so they have become tired of me. I am aware of not displaying stupid expressions, which can keep them at a distance’.*

Several of the participants expressed that during the course of treatment they had realized that the ACT team was not the only factor responsible for their adherence to treatment. It was also up to the clients themselves to accept the treatment, make the best of it and contribute to changes within themselves. ACT was experienced as both care and, to a lesser extent, control and surveillance. The experience was perceived as being dependent on the clients’ own attitudes, mainly being positive toward treatment. Furthermore, the service providers were perceived as being motivating and positive minded by the participants, and such attitudes were experienced as a driving force to success, on the condition that they themselves, as service users, were willing to strive for a better life.

## Discussion

This study aimed to explore the experiences of being recruited to and remaining in ACT by recovering clients diagnosed with SMI and concurrent substance use. The analysis showed that building trust through enduring involvement and receiving benefits in the initial phase were the most important factors for the initial acceptance of ACT by clients. The feeling of exclusiveness, with potential for improvement, the experience of ACT as a safety net and taking personal responsibility for treatment were stated as the most important factors for staying in treatment. Mostly, the participants pointed to the impact of the service providers’ attitudes and actions both on recruitment and on staying in treatment; however, they also emphasized their own contributions as being important, especially for remaining in treatment.

### Initial engagement

There is evidence that engagement is not related to a client’s level of symptoms or degree of insight [26]. Therefore, clients should not be considered as being too unsuitable or lacking insight to such a degree that it is not worth attempting to engage them. Clients who actively resist service providers’ attempts to engage them might be perceived as counteracting treatment and the therapeutic process. Conversely, this can be a sign of resilience and a determined holding on to a personal perspective. Thus, although other services have found these individually minded people to be “problematic”, ACT often views them as being determined and strong individuals [27]. Although they shared the label of improving clients, most participants in our study expressed attitudes of both scepticism and relief when first approached by ACT. This is reasonable, taking into account their experiences of limited success in traditional psychiatric and substance-use treatment.

A model of a hierarchy of needs has been developed to try to understand the mechanisms involved in work with clients with SMI and their initial engagement. Needs located at the bottom of the hierarchy, such as food, shelter and physical security, are highlighted as target priorities [28]. This implies that if ACT can assist someone in sorting out their benefits or housing problems, the person will then be able to think about engaging in other activities, such as social, occupational and psychotherapeutic interventions. The fact that the participants in our study viewed service providers as displaying a caring manner and stable housing as a platform for developing trust toward the service providers can be perceived as supportive of such a model. As indicated by our study participants, being overall positive toward treatment implies that successful engagement can render clients more accessible to psychotherapeutic intervention. However, available research indicates that many ACT teams do not provide services that entail psychotherapy, or if they do, only a minority of their clients actually receive such therapy [12, 29, 30].

It usually takes about 1 year for an effective therapeutic relationship to become established in ACT, and 18 months is a key point by which positive changes should be expected [31]. Even though the mean duration of treatment in ACT for the participants in our study was 22 months, some of the participants had been in treatment for less than 18 months. Furthermore, the service providers, although enthusiastic, were inexperienced in the ACT setting. The participants in our study valued highly the service providers who were persistent in their efforts to establish the initial contact, even if a majority of the participants were reluctant at first. Their attitude toward the service providers and treatment in general became more positive after a trusting relationship had developed. This finding is in line with one qualitative study that examined nurses’ experiences of the ACT engagement process [32]. Findings indicate that, in order to recruit clients into ACT effectively, the time spent connecting with the client and working at the client’s pace and level of persistence should be emphasized. Another qualitative study focused on the initial engagement of “hard-to-reach” clients by a community mental health team that worked partly in an assertive outreach manner [33]. That study described both a client group that was largely invisible to local services and the daily experiences of treatment providers while attempting to engage such clients. The authors found that long, slow and persistent contact was a core feature of the work. Although neither of the studies mentioned above considered the client perspective, the findings that reflect the professional stance are similar to those of our study regarding the importance of time and persistence. In addition, the participants in our study viewed benefits as being equally important in their decision to accept ACT at first. One explanation for this result could be that the cited study [33] was aimed at examining the barriers to collaborative work and relied only partly on the ACT model regarding the treatment approach; therefore, the benefit issue was not prevalent in that study. A study of clients’ perceptions of first-year experiences of participation in ACT in which the majority of participants were substance users found that service delivery in a caring manner and with persistence and a service that aids in practical matters were the most important factors for engagement [18]. The findings of that study are similar to the results of our study regarding initial engagement in ACT.

The key principle of the latest version of the ACT model regarding client engagement is the establishment of a collaborative and nonconfrontational joint-decision approach. The aim of this method is to enhance clients’ intrinsic motivation to access the services of the team [1, 12]. The importance of assistance regarding practical matters serving as a gateway to accepting treatment initially was salient in our participants’ statements. Assistance in attaining stable housing and help with work possibilities and social benefits were also important. Some participants described this assistance as an aid in self-realization and as the foundation for trusting the service providers. This aspect seemed prevalent regardless of whether they were recruited to ACT from institutional care, were receiving traditional community mental health services, or were receiving no treatment at all. The second step of the ACT model in delineating procedures for the engagement of clients focuses on limit-setting interventions. This implies approaches aimed at ensuring that treatment needs are met, so that the risk of harm to self or others is minimized [1, 12]. With the exception of one participant who received medication via coercive means and another participant who had been hospitalized involuntarily between the interviews, none of the participants had experiences of limit-setting interventions since joining ACT. Nevertheless, some participants expressed concern regarding whether poor compliance with medication regimes could lead to a more restrictive practice by ACT. Criticism has been raised of ACT as being paternalistic and coercive [34, 35], but most available research reports ACT as being non-coercive [36, 37]. In addition, clients report generally high levels of satisfaction with ACT, and do not characterize themselves as being coerced [15, 38, 39]. The results of our study are well in line with those of the latter studies. Overall, our study participants clearly expressed that motivational interventions were used far more in the engagement phase compared with limit-setting interventions.

### Maintained engagement

One study [40] introduced a measure of continued care based on the findings of an ethnographic study [41] that used community mental health centres and included the perspectives of both service providers and clients. The findings showed that knowledge, flexibility, availability, co-ordination and management of transition were conceptual mechanisms that were involved in promoting continuity of care. Compared with the perspectives of the clients included in our study, both knowledge and flexibility can be seen as overlapping with the sub-theme of exclusiveness in our study. The conceptual mechanism of availability represents, in a broad sense, the same as the finding of ACT as a safety net, according to the clients’ awareness of having someone to back them up.

The attachment theory argues that psychological development and functioning are affected by our earliest attachment to caregivers [42, 43]. Mental illness is likely to be a potent stimulator of attachment behaviour because of the threat to internal as well as external safety [44]. Similarly, people with substance-use problems often suffer from relational problems, regardless of whether they are the cause or consequence of the substance use [45]. When service providers function as attachment figures, they contribute to modulating anxiety and providing a secure base. This process is described as an interaction toward which both the service provider and the client contribute. Examples of this in our study were some participants’ attitudes in the form of reciprocal acts toward the service providers, because the clients felt grateful after being treated well. This resulted in feelings of commitment to following up on appointments, behaving well and being positive overall toward treatment. Furthermore, this was also understood as a way of helping themselves. These attitudes can also be perceived as a success in enhancing the clients’ intrinsic motivation to access the services of the team, according to the ACT model [1, 12]. The importance of a safety net was expressed both in the case of worsening SMI and in the case of escalating substance use. These aspects were more prominent in our study than they were in comparable studies [17, 18]. One reason for this discrepancy could be that persons who are diagnosed with SMI and also use substances are more prone to experiencing “ups and downs” than persons who are not using substances. The two studies cited above both included cohorts in which most, but not all, individuals engaged in substance use. The aim of one of those case studies was to determine how ACT contributes to the improvement of clients who receive the service, from the perspectives of both the clients and their service providers [17]. The main findings of that study were the persistence demonstrated by service providers in engaging their clients and the trust that the clients developed in their service providers. Similarly, our study identified exclusiveness as an important factor, including elements of commonplace communication with service providers and being treated respectfully, hence facilitating a trusting relationship.

The majority of the participants in our study welcomed the service providers into their home once a trusting relationship had been established. The participants acknowledged the delivery of services to their home or surroundings, and cited breaking down their isolation and the availability of service providers as important factors in this process. Because of the short duration of treatment in our study, it was difficult to illustrate the importance of the outreach working model regarding drop-out rates. Visiting clients at home provides the opportunity for more frequent meetings with the clients, who otherwise might not come to appointments, either because they are too disorganized or because they lack an understanding of their need for treatment. There is research indicating that this can increase engagement [46]. The willingness of service providers to meet the clients in their home environment, which is perceived as both a strength and a limitation, has been stressed in several studies [44, 47].

### Clinical implications

Service providers need to recognize that clients with SMI who use substances may have attachment difficulties and that they may conceptualize service providers as family or friends after the initial phase of ACT. Therefore, longitudinal treatment and the inclusion of several service providers in the ACT team are needed. It is important to consider that clients with SMI and concurrent substance use may be more vulnerable, and more in need of ACT as a safety net, than non-using clients. It appears important to evaluate continually the use of home visits, and to work collaboratively with the clients to establish suitable venues and schedules. After the initial phase, service providers should consider the gradual transfer of personal responsibility to the client and evaluate this process together with the client in question. One implication of the results of the present study is that the service providers have to prove that they can be trusted in the initial phase of the client’s contact with the team. Trust can be established over time by flexible and highly motivated service providers who place emphasis on relational aspects and aid in practical matters. Receiving benefits from ACT is of importance when accepting this service. The introduction of ACT when new housing is available after hospitalization is one option; however, it is important to know that practical help, social contact and support are also experienced as benefits. As suggested by the results of this study, the feeling by clients with SMI and concurrent substance use that service providers in ACT believe they can improve their client’s quality of life, is of importance for feeling exclusive, having hope for the future and remaining in treatment. Services provided by ACT should also be established to meet clients’ needs for psychotherapeutic intervention when they are successfully engaged with the team. Looking ahead, the client’s awareness of supportive and emphatic service providers can prevent drop-out and facilitate strategies for future discharge from treatment. More qualitative research on clients’ perspectives of engagement in treatment settings other than ACT is necessary. Moreover, there is a need for additional studies to assess how clients with severe mental illness and concurrent substance use view inclusion in treatment and to determine the factors that are important for them to remain in treatment.

### Limitations

The use of a sample of clients in recovery who were mainly positive toward ACT, and thus not representative of the full range of clients enrolled in ACT, may limit the transferability of our results, as may the interviewers’ focus on factors that influenced improvement and well-being. The Norwegian ACT teams have only been established recently. They consist of highly motivated service providers who are eager to work according to a new treatment model, and few clients were included in the phase of establishment of the teams. This could also explain some of the mainly positive experiences described by the participants in our study. Most of the interviews took place in meeting rooms in convenient locations provided by the ACT team. Therefore, the participants might have expressed themselves in mainly positive terms regarding their experience with ACT based on the setting in which the interviews took place. Furthermore, having service providers attend some of the interviews may have influenced the issues that were brought up. This study was not designed to attempt to evaluate the ACT model; rather, it was meant to explore the participants’ experiences, with a main focus on relational factors.

## Conclusions

The findings of this explorative study, which focused on participants with SMI and concurrent substance use, highlighted the factors that were most important for the successful recruitment and adherence of clients to ACT. Building trust through enduring involvement and receiving benefits were most important for the acceptance of ACT by the clients during the initial engagement. A feeling of exclusiveness, the perception of ACT as a safety net and the clients’ own personal responsibility for taking part in the treatment were stated as the most important factors for staying in treatment. The data support the findings of other qualitative studies regarding the importance of enduring involvement, and partly support their findings regarding the importance of receiving benefits for initial engagement. The importance of a feeling of exclusiveness and the perception of ACT as a safety net to maintain engagement was reported by other studies, whereas the importance of personal responsibility on behalf of the clients was not described in other reports. These results can be interpreted as supportive of ACT as a treatment model that is well suited to the engagement of clients with SMI and concurrent substance use.
